# Joint modelling of longitudinal lipids and time to coronary heart disease in the Jackson Heart Study

**DOI:** 10.1186/s12874-020-01177-7

**Published:** 2020-12-03

**Authors:** Wondwosen Kassahun-Yimer, Karen A. Valle, Adebamike A. Oshunbade, Michael E. Hall, Yuan-I. Min, Loretta Cain-Shields, Pramod Anugu, Adolfo Correa

**Affiliations:** 1 Department of Data Science, University of Mississippi Medical Center, 2500 N State St, Jackson, 39216 MS USA; 2 Department of Medicine, University of Mississippi Medical Center, 2500 N State St, Jackson, 39216 MS USA; 3 Department of Medicine, University of California San Francisco, 1701 Divisadero St, San Francisco, 94115 CA USA

**Keywords:** Joint modeling, Multivariate longitudinal, Survival data, Correlated responses

## Abstract

**Background:**

Multiple longitudinal responses together with time-to-event outcome are common in biomedical studies. There are several instances where the longitudinal responses are correlated with each other and at the same time each longitudinal response is associated with the survival outcome. The main purpose of this study is to present and explore a joint modeling approach for multiple correlated longitudinal responses and a survival outcome. The method will be illustrated using the Jackson Heart Study (JHS), which is one of the largest cardiovascular studies among African Americans.

**Methods:**

Four longitudinal responses, i.e., total cholesterol (TC), high density lipoprotein (HDL) cholesterol, triglyceride (TG) and inflammation measured by high-sensitivity C-reactive protein (hsCRP); and time-to-coronary heart disease (CHD) were considered from the JHS. The repeated lipid and hsCRP measurements from a given subject overtime are likely correlated with each other and could influence the subject’s risk for CHD. A joint modeling framework is considered. To deal with the high dimensionality due to the multiple longitudinal profiles, we use a pairwise bivariate model fitting approach that was developed in the context of multivariate Gaussian random effects models. The method is further explored through simulations.

**Results:**

The proposed model performed well in terms of bias and relative efficiency. The JHS data analysis showed that lipid and hsCRP trajectories could exhibit interdependence in their joint evolution and have impact on CHD risk.

**Conclusions:**

We applied a unified and flexible joint modeling approach to analyze multiple correlated longitudinal responses and survival outcome. The method accounts for the correlation among the longitudinal responses as well as the association between each longitudinal response and the survival outcome at once. This helps to explore how the combination of multiple longitudinal trajectories could be related to the survival process.

## Background

Longitudinal data together with time-to-event measurements are common in biomedical studies. In cardiovascular researches, for example, as we focus in this paper, lipid levels, i.e., total cholesterol (TC), low density lipoprotein (LDL) cholesterol, high density lipoprotein (HDL) cholesterol and triglyceride (TG) could be measured repeatedly over time along with time-to-event data such as time to coronary heart disease (CHD). Abnormalities in LDL cholesterol, HDL cholesterol, TC and TG levels have all been linked to increased risk for CHD. Furthermore, inflammation measured by high-sensitivity C-reactive protein (hsCRP) correlates with CHD status. As people age, body mass and composition as well as physical activity levels and diet tend to change which are often associated with changes in lipid levels.

There is an extensive literature available on separate analyses of longitudinal measurement data and time-to-event data including the popular linear mixed effects model for the former [1, 2], and parametric (Weibull model or exponential model) or semiparametric (Cox) proportional hazards model for the latter [3]. However, separate analyses could be inappropriate in some applications as health status (for example, risk to CHD) is likely correlated with longitudinal lipid level trajectory. Furthermore, separate analysis may not adequately address the underlying research questions. Wang and Taylor [4] include the longitudinal marker as a time-dependent covariate in the (proportional hazards) survival model. It has been shown, however, that including longitudinal measurements as time-varying covariate may be inappropriate as the measurements could be subject to measurement error [5]. Several approaches of joint modeling of a longitudinal continuous response and a time-to-event outcome have been available. Tsiatis and Davidian [6] proposed a two-stage joint model in application to longitudinal CD4 count data as surrogate marker to HIV/AIDS survival whereby a repeated measures random components model is considered in the first stage for the longitudinal CD4 count data while a Cox model is employed in the second stage for estimating the parameters. Henderson et al. [7] proposed a flexible joint modelling framework such that the longitudinal and survival processes are connected via a latent bivariate Gaussian process. A fully Bayesian version of this approach, implemented via Markov chain Monte Carlo (MCMC) methods is presented in Guo and Carlin [8]. Ibrahim et al. [9] applied such joint modeling and explored why it is needed in cancer research. A joint model for longitudinal and survival data with nonparametirc multiplicative random effects approach has been studied by Ding and Wang [10].

The majority of the joint models in the literature focus on a single longitudinal response and a survival outcome. Little attention has been given to modeling multiple longitudinal responses and a survival process simultaneously. Flexible semiparametric multivariate joint model that relate multiple longitudinal outcomes to a time-to-event have been proposed [11, 12] with emphasis on the association between the longitudinal data and survival outcome. Such multivariate joint analysis is also needed when one aims to explore not only the association between an individual longitudinal outcome and a survival process but also the correlation between the multiple longitudinal responses. In the Jackson Heart Study (JHS), described in “Methods” section, longitudinal HDL, TC, TG and hsCRP measurements taken from each subject repeatedly over time are subject to measurement error, potentially correlated with each other, and at the same time each of these lipid and hsCRP profiles is likely associated with the subject’s risk for CHD. It is then useful to assess these features by studying their association and simultaneous covariate effects, which can be addressed in joint modelling context. However, joint modeling of such multivariate longitudinal responses and a survival outcome poses computational challenges due to the high dimensionality in the random effects model.

This paper aims to present a joint modeling approach for multiple longitudinal responses and a survival data. To deal with the high dimensionality due to multiple longitudinal components, a pairwise model fitting approach that was developed in the context of multivariate Gaussian random effects is adopted [13].

The rest of the paper is organized as follows. In “Study data” section, a motivating dataset is described with analysis reported in “Results” section. “Review of basic models” section focuses on brief review of models for separate and joint analysis of a continuous longitudinal response and a survival outcome. “Multivariate joint model” section focuses on a joint model for multivariate continuous longitudinal responses and a time-to-event outcome, followed by a likelihood based estimation approach along the lines of Fieuws and Verbeke [13] in “Estimation” section. “Simulation” section presents results of a simulation study. Discussion and concluding statements are given in “Discussion” and “Conclusion” sections, respectively.

## Methods

### Study data

The Jackson Heart Study (JHS) is a longitudinal community-based study that investigates the causes of cardiovascular disease among African Americans. JHS participants were recruited from urban and rural areas from the three counties that make up the Jackson metropolitan area (Hinds, Madison, and Rankin). The 5,306 enrolled participants received three clinical examinations (Exam 1, 2000-04; Exam 2, 2005-08; Exam 3, 2009-13) that generated a plethora of longitudinal data including, but not limited to, cardiovascular disease risk factors, socio-economic information and biochemical analytes. In addition to the clinic exams, JHS has kept track of the occurrence of events of interest (i.e. coronary heart disease, abbreviated as CHD) through adjudicated events. Details on the design methods and data collection of the JHS can be obtained from Taylor et al. [14] and Carpenter et al. [15].

**Fig. 1:**
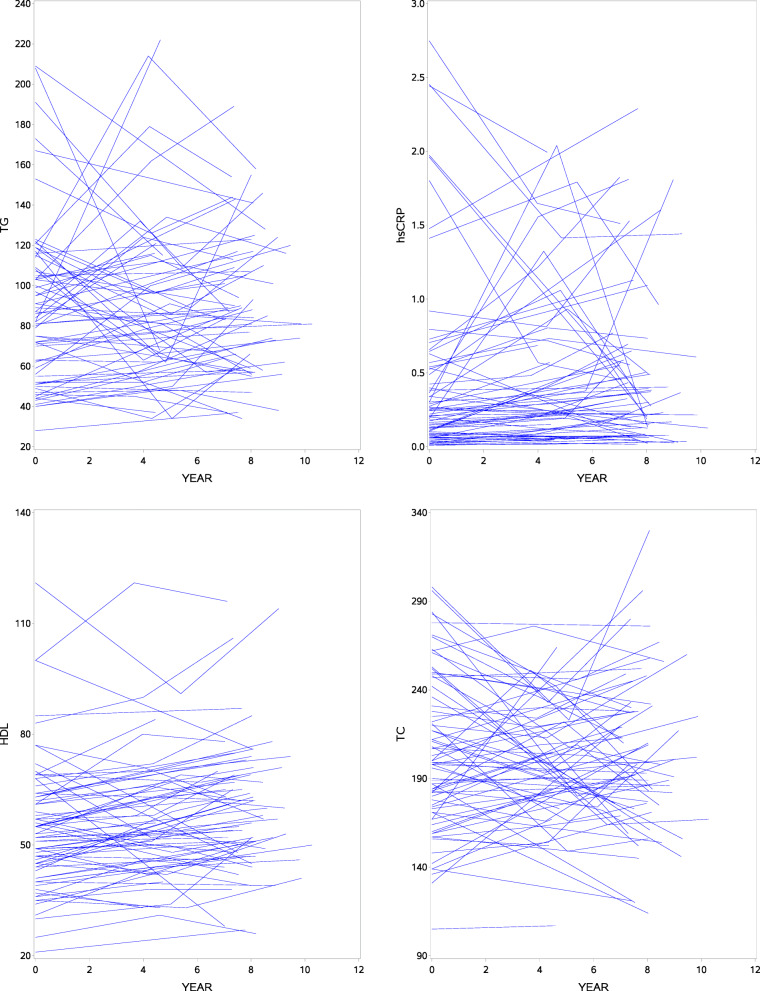
Individual profiles plot of triglyceride (TG), high-sensitivity C-reactive protein (hsCRP), high density lipoprotein (HDL) and total cholesterol (TC) for a random sample of 100 participants

**Fig. 2:**
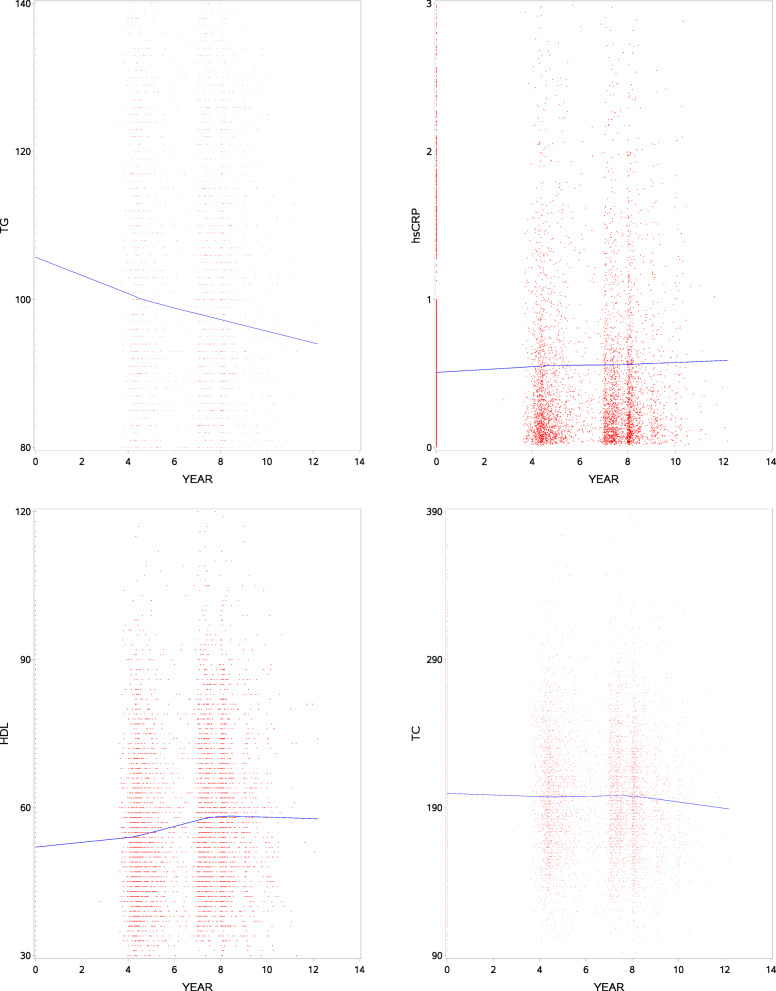
Average trajectory of triglyceride (TG), high-sensitivity C-reactive protein (hsCRP), high density lipoprotein (HDL) and total cholesterol (TC) for all participants

This paper uses data from two sources of JHS: (1) the longitudinal clinical examinations where sex, diabetes status, hypertension status, statin use, body mass index, inflammation, and lipid levels are extracted from; and (2) the annual follow-up surveys where Coronary Heart Disease (CHD) adjudicated events through 2016 are obtained. We allow for right censoring, i.e., those who experienced CHD since the baseline year (2000) were considered as incident cases and those who have died, withdrew from the study or have not seen CHD until 2016 are treated as censored. The time variable (event or censoring) begins from the baseline year and we haven’t assumed truncation as present. These variables are summarized in Table 1 by CHD status across the three measurement occasions where count and percentages are used for categorical variables and means and SDs for continuous variables. There are some notable highlights. Of the 4232 participants who have lipids and hsCRP measurements available at baseline, 236 (5.6%) experienced CHD event with a median survival time of 13.8 years. The mean age at baseline for non-CHD was 53.2 ±12 years while 62.0 ±10 years for those who had CHD. The proportion of diabetes as well as hypertension is higher in the CHD incident cases as compared to those without CHD across all visits. The mean HDL seems to increase over time, though the magnitude of mean level in non-CHD participants appears slightly higher when compared across the respective visits. The mean TG, however, tends to decline in both groups and non-CHD participants appear to have lower values. Incident cases are more likely to be men. Individual profiles plots of TG, hsCRP, HDL and TC for a random sample of one hundred participants are shown in Fig. 1. Clearly, there is evidence of between-subjects variability both at baseline and over time. On the other hand, Fig. 2 shows the average profiles plots of TG, hsCRP, HDL and TC for the full cohort. The average TG trajectory tends to decline while hsCRP and HDL seem to suggest an increasing average trend. TC appears to be more or less stable. A formal statistical test is needed to see the significance of these comparisons which will be dealt with later in “Results” section.

**Table 1:** Participants Characteristics by coronary heart disease status and visit through 2016, Jackson Heart Study

	**Incident coronary heart disease**			**No incident coronary heart disease**		
**Variables**	**Visit1**	**Visit2**	**Visit3**	**Visit1**	**Visit2**	**Visit3**
	**(n=236)**	**(n=82)**	**(n=120)**	**(n=3996)**	**(n=1929)**	**(n=2564)**
Men	99 (42.0%)	31 (37.8%)	44 (38.3%)	1417 (35.5%)	640 (33.2%)	883 (34.4%)
Diabetic	93 (39.4%)	32 (39.0%)	47 (39.2%)	676 (16.9%)	447 (23.2%)	678 (26.4%)
Hypertension	196 (83.1%)	73 (89.0%)	111 (92.5%)	2070 (51.8%)	1314 (68.1%)	1882 (73.4%)
Statin	61 (25.9%)	40 (48.8%)	69 (57.5%)	427 (10.7%)	547 (28.4%)	938 (36.6%)
BMI	31.22 (6.8)	31.73 (6.7)	31.04 (7.1)	31.78 (7.2)	32.31 (7.2)	32.31 (7.3)
HDL	50.69 (15.2)	51.64 (15.1)	56.93 (17.6)	52.05 (14.4)	54.67 (15.1)	58.38 (15.8)
TC	205.89 (43.9)	198.79 (49.0)	197.52 (51.3)	199.33 (39.5)	197.04 (40.5)	197.91 (39.2)
TG	127.40 (125.3)	113.12 (96.2)	106.55 (66.1)	104.45 (76.8)	99.23 (89.0)	96.95 (55.8)
hsCRP	0.61 (0.9)	0.84 (1.8)	0.51 (0.7)	0.50 (0.9)	0.55 (0.7)	0.56 (0.9)

The numbers presented are mean (SD) for continuous variables and frequency (percent) for categorical variables

The Jackson Heart Study provides a unique opportunity to study the longitudinal trajectory of lipids and hsCRP and incident CHD. Therefore, we will assess the association between the longitudinal lipids and hsCRP with incident CHD as well as the correlation between the longitudinal lipids and hsCRP measurements in African American participants.

### Review of basic models

In “Linear mixed model for longitudinal data”, we will briefly review the linear mixed model for continuous longitudinal data, followed by a basic parametric model for survival data in “Model for time-to-event data” sections. “Joint model” section focuses on a joint model for a longitudinal response and time-to-event outcome.

#### Linear mixed model for longitudinal data

Let $\phantom {\dot {i}\!}y_{i1}, y_{i2}, \ldots y_{i n_{i}}$ be longitudinal measurements from the *i*^*th*^ subject at times $\phantom {\dot {i}\!}t_{i1}, t_{i2}, \ldots t_{i n_{i}}$. The model can be written as

1 $$ y_{{ij}}=\boldsymbol{{x^{\prime}_{1i}}}\left(t_{{ij}}\right) \boldsymbol{\beta} + {\boldsymbol{z}^{\prime}_{1i}}\left(t_{{ij}}\right) {\boldsymbol{b}_{1i}} + \varepsilon_{{ij}},  $$

where ***x***_1*i*_(*t*_*ij*_) are regressors corresponding to unknown regression coefficients ***β***,***b***_1*i*_ are random effects with design matrix ***z***_1_(*t*_*ij*_), and ***ε***_*i*_ are measurement errors. The subject-specific random effects ***b***_1*i*_ and measurement error ***ε***_*i*_ are independent and assumed to follow normal distribution, i.e., ***b***_1*i*_∼*N*(***0***,***D***), and ***ε***_*i*_∼*N*(***0***,***Σ***_*i*_). Details on linear mixed models can be found in Laird and Ware [1].

#### Model for time-to-event data

Suppose *s*_*i*_ denotes the survival time for subject $i,~i=1, 2, \dots, n.$ The event of interest may not be observed for some of the subjects until the end of the follow-up period and hence their event times are right censored. Let *c*_*i*_ be the censoring time for subject *i*. For an indicator function *δ*_*i*_=*I*(*s*_*i*_≤*c*_*i*_), it follows that, *δ*_*i*_=0 if the survival time for subject *i* is right censored and *δ*_*i*_=1 otherwise. For subject *i* the observed time-to-event data are (*t*_*i*_,*δ*_*i*_) where *t*_*i*_=min(*s*_*i*_,*c*_*i*_),*i*=1,2…,*n*.

Weibull and exponential models are commonly used parameteric models in survival data analysis. In a Weibull model the density for the survival time for the *i*^*th*^ subject is given by

2 $$ f(t_{i}) = \alpha {t_{i}}^{\alpha-1} \text{exp}\left(\lambda_{i} - \text{exp}(\lambda_{i}) {t_{i}}^{\alpha}\right),  $$

*α*>0,*λ*_*i*_ can be linked with covariates as *λ*_*i*_=***x***_2*i*_***ξ***, where ***x***_2*i*_ is the vector of covariates corresponding to the *i*^*th*^ observation and ***ξ*** is a vector of regression coefficients. The Weibull model in (2) reduces to the exponential model when *α*=1.

#### Joint model

The longitudinal response in “Linear mixed model for longitudinal data” and survival process in “Model for time-to-event data” sections can be correlated with each other. One possible route to study this association is through joint modeling, where the linear mixed model in (1) is linked with the survival model in (2) through shared random effects.

Assuming a linear mixed effects model for the longitudinal part and a Weibull model for the survival part, the joint distribution of the longitudinal response and the time-to-event outcome conditional on the random effects is:

$$\begin{array}{@{}rcl@{}} &&f_{i}\left(y_{{ij}}, t_{i}| {\boldsymbol{b}_{1i}}, {\boldsymbol{b}_{2i}},\boldsymbol{\beta},\boldsymbol{\xi}, \boldsymbol{\theta}\right)\\&&=\frac{1}{{(2\pi)}^{\frac{n_{i}}{2}}{|\Sigma_{i}|}^{\frac{1}{2}}} e^{\left\{-\frac{1}{2}\left(\boldsymbol{y_{i}}- \boldsymbol{{x^{\prime}_{1i}}} \boldsymbol{\beta} - {\boldsymbol{z}^{\prime}_{1i}} {\boldsymbol{b}_{1i}}\right)^{\prime}{\Sigma_{i}}^{-1}\left(\boldsymbol{y_{i}} - \boldsymbol{{x^{\prime}_{1i}}} \boldsymbol{\beta} - {\boldsymbol{z}^{\prime}_{1i}} {\boldsymbol{b}_{1i}}\right)\right\}} \\ &&\quad \times \left[\alpha {t_{i}}^{\alpha - 1} {e}^{\boldsymbol{{x^{\prime}_{2i}}} \boldsymbol{\xi} + {\boldsymbol{b}_{2i}}} \right]^{\delta_{i}} e^{{- t_{i}}^{\alpha} e^{\boldsymbol{{x^{\prime}_{2i}}} \boldsymbol{\xi} + {\boldsymbol{b}_{2i}}}}, \end{array} $$

where ***b***_2*i*_=***θb***_1*i*_,***b***_1*i*_ and ***b***_2*i*_ are latent zero-mean bivariate Gaussian processes corresponding to the longitudinal measurements and events, ***θ*** is vector of association parameters, and the rest of the quantities are as defined in “Linear mixed model for longitudinal data” and “Model for time-to-event data” sections. For random intercepts *b*_0*i*_ and random slopes *b*_1*i*_, for example, one possible model could be

$${\boldsymbol{z}^{\prime}_{1i}} {\boldsymbol{b}_{1i}}= b_{0i} + b_{1i} t_{{ij}},$$

$${\boldsymbol{b}_{2i}}= \theta_{1} b_{0i} + \theta_{2} b_{1i},$$

where *θ*_1_ and *θ*_2_ measure the association between the two submodels induced by the random intercepts and random slopes, respectively. Other ways of parametrization for the association of the two submodels can also be used as well [7]. Furthermore, ***b***_1*i*_=(*b*_0*i*_,*b*_1*i*_) is assumed to follow bivariate normal distribution, possibly correlated, with mean ***0*** and variance-covariance matrix *D* given by

3 $$  D= \left(\begin{array}{cc} d_{0}^{2}& \rho d_{0} d_{1}\\ \rho d_{0} d_{1}&d_{1}^{2}\\ \end{array} \right),  $$

where *ρ* is the correlation between the random intercepts and random slopes.

### Multivariate joint model

The joint mode in “Joint model” section can be extended to a multivariate setting where *k* longitudinal continuous responses can be simultaneously modeled with time-to-event data. A multivariate joint model formulation of the *k* longitudinal responses and the time-to-event outcome follows from combining each single joint model of the longitudinal continuous response and the time-to-event outcome via random effects that are assumed to follow multivariate normal distribution. The general expression for the resulting multivariate joint model can be given by

4 $$ f=\idotsint \prod\limits_{\ell=1}^{k} f (y_{\ell},t)|\boldsymbol{b_{\ell}}) \phi(\boldsymbol{b_{1}}, \ldots, \boldsymbol{b_{k}}) d\boldsymbol{b_{1}}\ldots d\boldsymbol{b_{k}},  $$

where *f*(*y*_*ℓ*_,*t*) is the single joint model corresponding to the *ℓ*^*th*^ continuous response and the time-to-event data as described in “Joint model” section and *ϕ* is a *k*-dimensional Gaussian density for the random effects (***b***_***1***_,…,***b***_***k***_), such that, for subject *i*, each ***b***_***ℓi***_ is shared between the linear mixed submodel corresponding to the *ℓ*^*th*^ longitudinal response and the time-to-event submodel through ***b***_1*i*_ and ***b***_2*i*_, respectively, as shown in “Joint model” section. The likelihood contribution of subject *i* is *l*_*i*_(***Y***_***1i***_,…,***Y***_***ki***_,*t*_*i*_|***Λ***), where ***Λ*** is the vector of fixed effect and covariance parameters in the joint model. The full multivariate joint model in (4) is complex due to the high dimensionality in the random effects and inference for ***Λ*** is not feasible in the standard statistical softwares. An estimation approach to overcome this computational challenge and reduce the dimensionality along the lines of Fieuws and Verbeke [13] will be the topic of “Estimation” section.

### Estimation

We employ the pairwise bivariate fitting to reduce the high dimensionality of (4) and estimate the model parameters in ***Λ*** [13]. By pairwise fitting we mean that bivariate joint models of all possible pairwise combinations of the longitudinal continuous responses and a time-to-event data are fitted and duplicate parameter estimates are averaged across all pairs. Consider *k* random-intercept models that are specific to *k* single joint models where each joint model represents a longitudinal continuous responses and time-to-event outcome as described in “Joint model” section. The possible number of pairs out of the *k* continuous responses is $P=\frac {k(k-1)}{2}$. For subject *i*, consider a vector of the *r*^*th*^ and *s*^*th*^ bivariate longitudinal responses and the time-to-event outcome (***Y***_***ri***_^′^,*t*_*i*_) and (***Y***_***si***_^′^,*t*_*i*_), respectively, where $\boldsymbol {Y_{{ri}}}=\left (y_{ri1}, \ldots, y_{ri n_{{ri}}}\right)$ and $\boldsymbol {Y_{{si}}}=\left (y_{si1}, \ldots, y_{si n_{{si}}}\right), r=1, \ldots, k-1, s=r+1,\ldots, k.$ We assume that (***Y***_***ri***_,*t*_*i*_) and (***Y***_***si***_,*t*_*i*_) are conditionally independent given ***b***_***i***_=(*b*_*ri*_,*b*_*si*_)^′^, where ***b***_***i***_∼MVN(**0**,*D*) is vector of random effects corresponding to the *r*^*th*^ and *s*^*th*^ bivariate joint models with mean **0** and covariance matrix *D*.

The likelihood to be maximized corresponding to the *r*^*th*^ and *s*^*th*^ bivariate joint model of two longitudinal continuous responses the time-to-event data takes the form

5 $$ \begin{aligned} {}\prod\limits_{i=1}^{N}\int\int&\left\{ \prod\limits_{j=1}^{n_{{ri}}}f \left(y_{{rij}}, t_{i}|\boldsymbol{b_{{ri}}}\right)\right\} \\\times&\left\{ \prod\limits_{j=1}^{n_{{si}}}f \left(y_{{sij}}, t_{i}|\boldsymbol{b_{{si}}}\right)\right\} \phi\left(\boldsymbol{b_{{ri}}}, \boldsymbol{b_{{si}}}\right) d\boldsymbol{b_{{ri}}}d\boldsymbol{b_{{si}}}, \end{aligned}  $$

where *f* and *ϕ* are as defined in (4) and *N* is the total number of subjects. Fitting these bivariate models is computationally feasible as there are only two correlated random effects. The loglikelihood across all possible pairs can be given as ${\sum \nolimits }_{p=1}^{P} l\left (\boldsymbol {Y_{p}} | \boldsymbol {\Lambda }^{\prime }_{p}\right)$, where *p*=1,…,*P*, and $\boldsymbol {\Lambda }^{\prime }_{p}$ is the vector of parameters corresponding to the *p*^*th*^ bivariate joint model. All pair-specific parameter vectors $\boldsymbol {\Lambda }^{\prime }_{p}$ can be combined and the so resulting vector of parameters is denoted by ***Λ***^′^. Assuming that $\widehat {\boldsymbol {\Lambda }^{\prime }_{p}}$ maximizes $l\left (\boldsymbol {Y_{p}} | \boldsymbol {\Lambda }^{\prime }_{p}\right), \widehat {\boldsymbol {\Lambda }^{\prime }}$ would maximize *l*(***Λ***^′^). An estimate for each parameter in ***Λ*** of the full multivariate joint model defined in “Multivariate joint model” section is obtained by averaging all pair-specific estimates in $\widehat {\boldsymbol {\Lambda }^{\prime }}$. The resulting averages are asymptotically normal. However, the standard errors can’t be directly computed by simple averaging of their corresponding pair-specific estimates. Details on the averaging of pair specific estimates and expression of the asymptotic normality of ***Λ***^′^ follows in a similar way as the multivariate linear mixed model and multivariate semi-continuous data and can be obtained in Fieuws and Verbeke [13]. This approach can be well generalized to random intercepts and random slopes model of course with additional computational complexity. Each bivariate pair consists of a longitudinal response and a time-to-event outcome as shown in (5) does not have a closed form solution and is fitted numerically with the SAS procedure NLMIXED using adaptive Gaussian quadrature based on quasi-Newton optimization technique. Other flexible non-linear optimizers can be employed as well. A SAS macro has been used to fit the *P* bivariate models and combine the results.

## Results

We analyze the JHS data introduced in “Study data” section. A multivariate joint model of “Multivariate joint model” section will be fitted for the longitudinal measurements of high density lipoprotein (HDL) cholesterol, triglyceride (TG), total cholesterol (TC), high-sensitivity C-reactive protein (hsCRP), and time to coronary heart disease (CHD) to capture the association between each of these longitudinal response and the time-to-CHD as well as the correlation between HDL, TG, TC and hsCRP. Log-transformation was applied to the four longitudinal responses based on earlier exploratory analysis. Let *y*_*ℓij*_,*ℓ*=1,…,4 be the *ℓ*^*th*^ log-transformed continuous response for subject *i* at time *j* and we model the mean *μ*_*ℓij*_ as

$$\begin{array}{*{20}l}\mu_{\ell ij}&=\beta_{\ell 0}+\beta_{\ell 1}A_{i}+\beta_{\ell 2} M_{i}+\beta_{\ell 3} D_{{ij}}+ \beta_{\ell 4} H_{{ij}}\\&\quad+\beta_{\ell 5} T_{{ij}}+\beta_{\ell 6} BMI_{{ij}}+\beta_{\ell 7} ST_{{ij}}+\beta_{\ell 8} SM_{i} +b_{\ell i}, \end{array} $$

where *A*_*i*_ baseline age for subject *i*, *M*_*i*_ a gender indicator for subject *i* coded as (1: male; 0: female), *D*_*ij*_ is a diabetic status indicator for subject *i* at time *j* coded as (1: diabetic; 0: non-diabetic), *H*_*ij*_ is a hypertension status indicator for subject *i* at time *j* coded as (1: hypertensive; 0: non-hypertensive); *T*_*ij*_ is the *j* measurement time for subject *i* in years from baseline; *BMI*_*ij*_ is body mass index for subject *i* at time *j*, *ST*_*ij*_ is a statin medication indictor for subject *i* at time *j* coded as (1: medication; 0: no medication), *SM*_*i*_ is current smoking indicator for subject *i* at baseline coded as (1: smoker; 0: non-smoker) and *b*_*ℓi*_ are subject specific random intercepts for the *ℓ*^*th*^ continuous response.

Turning to the time-to-CHD, we consider an exponential submodel in each joint model with the *ℓ*^*th*^ continuous response, and further the hazard at time *t* is modeled as,

$$\begin{array}{*{20}l}\lambda_{\ell i} (t)&=\text{exp}\left(\xi_{\ell 0}+\xi_{\ell 1}A_{i}+\xi_{\ell 2} M_{i}+\xi_{\ell 3} D_{i}+ \xi_{\ell 4} H_{i}\right.\\&\quad\left.+\xi_{\ell 5} BMI_{i}+\xi_{\ell 6} ST_{i}+\xi_{\ell 7} SM_{i} + \theta_{\ell} b_{\ell i}\right), \end{array} $$

where *θ*_*ℓ*_ captures the association between the linear mixed submodel and the exponential submodel induced by the random effects. The correlation between any two pairs of longitudinal responses is captured by the correlation of the respective random intercepts.

Six pairs of bivariate models were fitted for the four responses using the pairwise fitting approach outlined in “Estimation” section. Results are shown in Table 2. Clearly, the estimated association parameter of TG and CHD is significant, suggesting evidence of strong negative association between the two submodels ($\hat {\theta }=-0.4473, p=0.009$), and this implies that high level of TG is associated with poor survival or shorter time-to-CHD. The same is true for the association between hsCRP and CHD ($\hat {\theta }=-0.2504, p=0.005$). On the contrary, the estimate corresponding to HDL ($\hat {\theta }=1.0472, p=0.002$) is positive and significant suggesting high HDL level is associated with better survival. On the other hand, the association parameter estimate corresponding to TC provides no evidence of association between total cholestol and CHD (*p*=0.151). Consider testing hypotheses about the four association parameters jointly. One can employ a multivariate Wald test for the hypothesis *H*_0_=**L**(***θ***)=0, where **L** is a 4 ×4 identity matrix corresponding to $\widehat {\theta }_{\ell }, \ell =1, \ldots, 4$. Thus, $W^{2}=\left (\mathbf {L} \boldsymbol {\widehat {\theta }}\right)^{\prime } {\left (\mathbf {L}\text {COV}(\widehat {\boldsymbol {\theta }}) \mathbf {L}^{\prime }\right)}^{-1}\left (\mathbf {L}\widehat {\boldsymbol {\theta }}\right) \sim {{\chi }^{2}}_{(4)}$, where $\text {COV}\left (\widehat {\boldsymbol {\theta }}\right)$ is the 4 ×4 covariance matrix of $\widehat {\boldsymbol {\theta }}$ obtained from fitting the multivariate joint model. This test suggests joint significance of the association between the four longitudinal responses and CHD (*χ*^2^=18.72,*p*=0.0009). Another important output of this analysis is estimated correlation of the random intercepts. In this regard, we observe evidence of negative correlation between TG and HDL (−0.5026) as well as HDL and hsCRP (−0.1112), while positive correlation between TG and TC (0.4390), TG and hsCRP (0.1478), and HDL and TC (0.2235). However, there is no evidence of significant correlation between TC and hsCRP. The estimated standard deviation of the random effects are significant across all the responses, implying the presence of considerable between-subjects variability that needs to be appropriately accounted for. All the estimates corresponding to time in the linear mixed submodel $\left (\hat {\beta _{5}}\right)$ are significant, i.e., negative time effect for TG, but positive effect for HDL, TC and hsCRP, and this is inline with what has been observed from the average profiles plot in Fig. 2. Those who are in statin medication have lower TG, TC and hsCRP, while no signifcant assocation was observed between HDL and statin use. Smokers tend to have elevated mean TG and hsCRP as compared to non-smokers. Those who are diabetic have higher TG and hsCRP but lower HDL and TC. The estimates of covariate effects in the survival submodel are quite similar except some slight variation depending on which of the longitudinal responses of TG, HDL, TC or hsCRP was modeled in the linear mixed submodel, and this is expected as these lipid and hsCRP measures have different effects on CHD survival as demonstrated by the differences in the estimates of the association parameter (*θ*) as well as the random intercept standard deviations (*d*). CHD survival is estimated to be shorter among hypertensive patients. For example, looking the parameter estimate of HTN $\left (\hat {\xi _{4}}\right)$ correspond to TG, we observe that those who are hypertensive have about 60*%* (1−*e*^−0.9204^=1−0.3984) shorter survival time. Similarly, smokers, diabetic patients, male gender and older age have shorter survival or higher risk to CHD. But no significant association was observed between BMI and CHD survival. Likely due to the smaller number of repeated measurements per subject, incorporating an additional random slope term and association parameter *θ*_2_ as described in “‘Joint model” section did not improve model fit. This implies that the subject specific random intercepts are sufficient to describe the longitudinal aspect in these data.

**Table 2:** Parameter estimates and standard errors of multivariate joint modelling of Triglycerides (TG) with CHD, High density lipoprotein (HDL) with CHD, high-sensitivity C-reactive protein (hsCRP) with CHD, and Total cholesterol (TC) with CHD

		**TG**	**HDL**	**hsCRP**	**TC**
**Effect**	**Parameter**	**Estimate(SE)**	**Estimate(SE)**	**Estimate(SE)**	**Estimate(SE)**
Longitudinal					
Intercept	*β*_0_	3.7360 (0.0501)	4.1408 (0.0276)	-3.7758 (0.1158)	5.1797 (0.0194)
Age	*β*_1_	0.0054 (0.0006)	0.0022 (0.0003)	0.0058 (0.0003)	0.0025 (0.0003)
Men	*β*_2_	0.1343 (0.0155)	-0.2002 (0.0077)	-0.3700 (0.0322)	-0.0272 (0.0062)
Diabetes	*β*_3_	0.1018 (0.0140)	-0.0378 (0.0064)	0.0537 (0.0281)	-0.0172 (0.0059)
Hypertension	*β*_4_	0.0663 (0.0113)	0.0057 (0.0051)	0.1077 (0.0246)	0.0128 (0.0047)
Time	*β*_5_	-0.0047 (0.0010)	0.0138 (0.0004)	0.0226 (0.0023)	0.0028 (0.0004)
BMI	*β*_6_	0.0115 (0.0010)	-0.0085 (0.0006)	0.0666 (0.0024)	-0.0005 (0.0004)
Statin	*β*_7_	-0.0952 (0.0118)	0.0131 (0.0052)	-0.2588 (0.0258)	-0.1427 (0.0057)
Smoking	*β*_8_	0.1569 (0.0238)	-0.0209 (0.0118)	0.3624 (0.0530)	-0.0153 (0.0095)
Std dev. error	*σ*	0.2812 (0.0037)	0.1200 (0.0017)	0.6229 (0.0129)	0.1206 (0.0018)
Survival					
Intercept	*ξ*_0_	9.5160 (0.5302)	9.4283 (0.5296)	9.4111 (0.5241)	9.3920 (0.5116)
Age	*ξ*_1_	-0.0542 (0.0063)	-0.0525 (0.0063)	-0.0528 (0.0063)	-0.0530 (0.0063)
Men	*ξ*_2_	-0.4067 (0.1439)	-0.3893 (0.1439)	-0.3568 (0.1439)	-0.3820 (0.1416)
Diabetes	*ξ*_3_	-0.7477 (0.1396)	-0.7493 (0.1394)	-0.7683 (0.1397)	-0.7583 (0.1399)
Hypertension	*ξ*_4_	-0.9204 (0.1922)	-0.9219 (0.1911)	-0.9100 (0.1911)	-0.9415 (0.1917)
BMI	*ξ*_5_	0.0082 (0.0112)	0.0081 (0.0112)	0.0086 (0.0109)	0.0098 (0.0108)
Statin	*ξ*_6_	-0.3312 (0.1557)	-0.3830 (0.1530)	-0.3844 (0.1527)	-0.3360 (0.1563)
Smoking	*ξ*_7_	-0.7003 (0.1807)	-0.7022 (0.1792)	-0.6941 (0.1812)	-0.7026 (0.1815)
Std dev. RE	*d*	0.4061 (0.0070)	0.2153 (0.0029)	0.8643 (0.0141)	0.1598 (0.0027)
Assoc.	*θ*	-0.4473 (0.1705)	1.0472 (0.3404)	-0.2504 (0.0884)	-0.7682 (0.5354)
Correlation					
TG		1	-0.5026 (0.0159)	0.1478 (0.0190)	0.4390 (0.0185)
HDL			1	-0.1112 (0.0195)	0.2235 (0.0194)
hsCRP				1	0.0268 (0.0215)
TC					1

For comparisons purpose, separate joint model of “Joint model” section were fitted. The results are summarized in Table 3. When compared with their respective multivariate joint model estimates of Table 2, we notice slight differences in the association parameters estimates corresponding to TG and hsCRP as well as the intercept terms of the survival component. The rest of the parameter estimates appear to be similar. Furthermore, we fitted an exponential model for time-to-CHD by including only baseline values of lipids separately while adjusting for the same covariates of the survival submodel considered so far. The estimated regression coefficients (standard error) corresponding to baseline TG, HDL, hsCRP and TC are −0.2354 (0.0876),0.6206 (0.1834),−0.1674 (0.0421), and −0.5710 (0.2344), respectively. Comparing these results with the association parameter estimates of joint multivariate model in Table 2, we clearly observe a remarkable difference in modeling the longitudinal trajectories versus the baseline measurements of lipids. We will explore this further in “Simulation” section with simulation.

**Table 3:** Parameter estimates and standard errors of univariate joint modelling of Triglycerides (TG) with CHD, High density lipoprotein (HDL) with CHD, high-sensitivity C-reactive protein (hsCRP) with CHD, and Total cholesterol (TC) with CHD

		**TG**	**HDL**	**hsCRP**	**TC**
**Effect**	**Parameter**	**Estimate(SE)**	**Estimate(SE)**	**Estimate(SE)**	**Estimate(SE)**
Longitudinal					
Intercept	*β*_0_	3.7495 (0.0476)	4.1341 (0.0235)	-3.7712 (0.1023)	5.1841 (0.0192)
Age	*β*_1_	0.0053 (0.0006)	0.0022 (0.0003)	0.0059 (0.0013)	0.0025 (0.0002)
Men	*β*_2_	0.1334 (0.0150)	-0.1998 (0.0077)	-0.3733 (0.0322)	-0.0275 (0.0060)
Diabetes	*β*_3_	0.1030 (0.0131)	-0.0398 (0.0060)	0.0549 (0.0285)	-0.0159 (0.0054)
Hypertension	*β*_4_	0.0660 (0.0112)	0.0045 (0.0051)	0.1083 (0.0245)	0.0135 (0.0047)
Time	*β*_5_	-0.0048 (0.0010)	0.0139 (0.0004)	0.0224 (0.0022)	0.0027 (0.0004)
BMI	*β*_6_	0.0113 (0.0009)	-0.0083 (0.0004)	0.0664 (0.0020)	-0.0006 (0.0004)
Statin	*β*_7_	-0.0880 (0.0112)	0.0125 (0.0049)	-0.2526 (0.0244)	-0.1418 (0.0048)
Smoking	*β*_8_	0.1570 (0.0218)	-0.0206 (0.0111)	0.3516 (0.0469)	-0.0155 (0.0087)
Std dev. error	*σ*	0.2814 (0.0029)	0.1200 (0.0012)	0.6231 (0.0065)	0.1207 (0.0013)
Survival					
Intercept	*ξ*_0_	9.0594 (0.5993)	9.0681 (0.5947)	8.9882 (0.5899)	9.2058 (0.5926)
Age	*ξ*_1_	-0.0504 (0.0066)	-0.0496 (0.0066)	-0.0500 (0.0066)	-0.0515 (0.0066)
Men	*ξ*_2_	-0.3857 (0.1386)	-0.3534 (0.1382)	-0.3949 (0.1382)	-0.3633 (0.1384)
Diabetes	*ξ*_3_	-0.7587 (0.1430)	-0.7890 (0.1421)	-0.7463 (0.1429)	-0.7688 (0.1426)
Hypertension	*ξ*_4_	-0.9215(0.1828)	-0.9264(0.1834)	-0.8656(0.1814)	-0.9324(0.1834)
BMI	*ξ*_5_	0.0147 (0.0113)	0.0136 (0.0113)	0.0132 (0.0112)	0.0122 (0.0111)
Statin	*ξ*_6_	-0.3333 (0.1570)	-0.3864 (0.1548)	-0.3430 (0.1566)	-0.3192 (0.1586)
Smoking	*ξ*_7_	-0.6739 (0.1793)	-0.6862 (0.1791)	-0.2003 (0.2080)	-0.7033 (0.1792)
Std dev. RE	*d*	0.4052 (0.0058)	0.2151 (0.0028)	0.8639 (0.0124)	0.1595 (0.0024)
Assoc.	*θ*	-0.3980 (0.1736)	0.9992 (0.3273)	-0.3328 (0.0874)	-0.7620 (0.4817)

## Simulation

In this section, we report on a simulation study set up to examine the performance of the pairwise bivariate fitting in joint modeling of multiple longitudinal measurements and a time-to-event data. The estimates from the pairwise bivariate fitting will be compared with the full likelihood multivariate joint model in terms of bias and relative efficiency.

We randomly generated 1000 data sets for three correlated longitudinal responses *y*_*ℓij*_ and a time-to-event outcome *t*_*i*_ from the multivariate joint model of “Multivariate joint model” section for 1000 subject at 7 time points. The *ℓ*^*th*^ longitudinal data were generated as *y*_*ℓij*_=*β*_*ℓ*0_+*β*_*ℓ*1_*time*_*ij*_+*β*_*ℓ*2_*male*_*i*_+*b*_*ℓi*_+*ε*_*ℓij*_, *i*=1,…,1000;*j*=0,…,6;*ℓ*=1,2,3. Similarly, the time-to-event data corresponding to the *y*_*ℓij*_ is generated from Weibull(*α*_*ℓ*_,*μ*_*ℓi*_), where *μ*_*ℓi*_=*ξ*_*ℓ*0_+*ξ*_*ℓ*1_*male*_*i*_+*θ*_*ℓ*_*b*_*ℓi*_, and the true parameter values for the three responses were ***β***_1_=(3.7,−1,0.5)^′^,***β***_2_=(4,1,−0.5)^′^,***β***_3_=(5,1,−0.5)^′^,***ξ***_*ℓ*_=***ξ***=(5,−0.5), vector of association parameters of the longitudinal and time-to-event data ***θ***=(*θ*_1_,*θ*_2_,*θ*_3_)=(−0.8,0.6,−0.5),*α*_*ℓ*_=*α*=0.5, the residual errors *ε*_*ijℓ*_ are assumed independent, each generated from normal distribution with mean 0 and standard deviations 0.8, 1 and 1. The random intercepts (*b*_1*i*_,*b*_2*i*_,*b*_3*i*_) are assumed correlated and generated from multivariate normal distribution with mean **0**, standard deviations 0.7, 0.5, and 0.5, and correlations: *ρ*_12_=−0.5,*ρ*_13_=0.6 and *ρ*_13_=0.3.

The simulated data were analyzed by the multivariate joint model using pairwise bivariate fitting and full likelihood trivariate joint model. All the 1000 simulations converged in the pairwise fitting while 985 simulations do so in the full trivariate model, i.e., nearly 1.5*%* failed to converge in the latter. For comparison purposes, results of the 985 successful simulations of the full multivariate joint model and the same number of simulations from the pairwise fitting, based on simulation ID number, were used. The average estimated values from the pairwise bivariate fitting (*Mean*_*P*_) and full trivariate joint model (*Mean*_*F*_) are summarized in Table 4. All of the mean estimates from the pairwise fitting are close to the true values and nearly unbiased for the proposed approach. Furthermore, as shown in Table 5, the Monte Carlo standard errors from the pairwise fitting (*MC*.*SE*_*P*_) are close to those from the full trivariate joint model (*MC*.*SE*_*F*_) with their ratios range from 0.997 to 1.056, and this suggests that no considerable efficiency loss of the pairwise bivariate approach relative to the full likelihood multivariate model.

**Table 4:** Summary of simulation with pairwise fitting (*Mean*_*P*_) and full multivariate joint model (*Mean*_*F*_)

	**Response 1 (*Y***_**1**_**)**			**Response 2 (*Y***_**2**_**)**			**Response 3 (*Y***_**3**_**)**		
**Parameter**	**True**	***Mean***_***P***_	***Mean***_***F***_	**True**	***Mean***_***P***_	***Mean***_***F***_	**True**	***Mean***_***P***_	***Mean***_***F***_
*β*_0_	3.7	3.7000	3.7011	4	3.9998	3.9999	5	4.9987	5.0004
*β*_1_	-1	-1.0000	-1.0000	1	1.0000	0.9999	1	1.0002	1.0002
*β*_2_	0.5	0.5003	0.4980	-0.5	-0.4985	-0.4990	-0.5	-0.4978	-0.5011
*ξ*_0_	5	5.0137	5.0087	5	5.0111	5.0048	5	5.0096	4.9980
*ξ*_1_	-0.5	-0.5003	-0.4996	-0.5	-0.4988	-0.5006	-0.5	-0.5064	-0.4920
*σ*	0.8	0.8000	0.8001	1	1.0000	0.9997	1	1.0002	1.0002
*d*	0.7	0.6987	0.6985	0.5	0.4995	0.4995	0.5	0.4987	0.4988
*α*	0.5	0.5005	0.5009	0.5	0.5005	0.5012	0.5	0.5011	0.5014
*θ*	-0.8	-0.8046	-0.8333	0.6	0.6182	0.6054	-0.5	-0.4936	-0.5031
*ρ*_12_	-0.5	-0.5005	-0.5002						
*ρ*_13_	0.6	0.6020	0.6018						
*ρ*_23_	0.3	0.2999	0.3000

**Table 5:** Monte-Carlo standard error with pariwise fitting (*MC*.*SE*_*P*_) and full multivariate joint model (*MC*.*SE*_*F*_)

	**Response 1 (*Y***_**1**_**)**		**Response 2 (*Y***_**2**_**)**		**Response 3 (*Y***_**3**_**)**	
**Parameter**	***MC.SE***_***P***_	***MC.SE***_***F***_	***MC.SE***_***P***_	***MC.SE***_***F***_	***MC.SE***_***P***_	***MC.SE***_***F***_
*β*_0_	0.0369	0.0370	0.0332	0.0333	0.0332	0.0333
*β*_1_	0.0048	0.0048	0.0060	0.0060	0.0060	0.0060
*β*_2_	0.0482	0.0482	0.0396	0.0396	0.0396	0.0396
*ξ*_0_	0.1312	0.1313	0.1294	0.1291	0.1289	0.1287
*ξ*_1_	0.1837	0.1836	0.1812	0.1805	0.1804	0.1800
*σ*	0.0073	0.0073	0.0091	0.0091	0.0091	0.0091
*d*	0.0186	0.0186	0.0178	0.0178	0.0178	0.0178
*α*	0.0176	0.0176	0.0176	0.0176	0.0176	0.0176
*θ*	0.1412	0.1398	0.2251	0.2145	0.2229	0.2111
*ρ*_12_	0.0359	0.0359				
*ρ*_13_	0.0328	0.0328				
*ρ*_23_	0.0472	0.0472				

Furthermore, the simulated data were analyzed by Weibull model of “Model for time-to-event data” section to investigate the impact of omitting the longitudinal aspect as well as the correlation between the longitudinal responses. This was achieved by including only basline measurements of the longitudinal responses as covariates in the regression model for the time-o-event outcome. Three separate models were fitted as Weibull(*α*_*ℓ*_,*μ*_*ℓi*_), where *μ*_*ℓi*_=*ξ*_*ℓ*0_+*ξ*_*ℓ*1_*male*_*i*_+*θ*_*ℓ*_*y*_*ℓi*_,*y*_*ℓi*_ is baseline values for the longitudinal response *y*_*ℓij*_ and the rest of the quantizes remain defined earlier. The results are summarized in Table 6. Clearly, the impact of taking just the baseline values of *y*_*ℓi*_ and ignoring the longitudinal trajectories is remarkable. This can be clearly observed from the considerable bias of the regression coefficients including the association parameter *θ*.

**Table 6:** Summary of simulation with Weibull model

	**Response 1 (*Y***_**1**_**)**		**Response 2 (*Y***_**2**_**)**		**Response 3 (*Y***_**3**_**)**	
**Parameter**	**True**	***Mean***	**True**	***Mean***	**True**	***Mean***
*ξ*_0_	5	6.3444	5	4.5521	5	5.5113
*ξ*_1_	-0.5	-0.3247	-0.5	-0.4388	-0.5	-0.5447
*α*	0.5	0.4888	0.5	0.4968	0.5	0.4978
*θ*	-0.8	-0.3531	0.6	0.1169	-0.5	-0.1004

## Discussion

The method has been used to jointly analyze four multivariate longitudinal responses and a time-to-event outcome from a cardiovascular study on African Americans. The data analysis showed inverse relationship between HDL cholesterol and incidence of coronary heart disease, while elevated triglyceride level and high-sensitivity C-reactive protein are associated with increased risk for CHD. Duncan et al. [16] who studied association between longitudinal lipid trajectories and atherosclerotic cardiovascular disease (ASCVD) including CHD reported a similar finding on the association of HDL cholesterol and ASCVD. This is inline with Wilikins et al. [17] and Skrettebegr et al. [18]. While Duncan’s investigation did not find a significant association between triglyceride and ASCVD, we observe an association of triglyceride with CHD which is consistent with Sarwar et al. [19]. This is likely because in Ducan’s study ASCVDE includes CHD, stroke, or transient ischemic attack, and intermittent claudiction while the present study and Sarwar’s investigation focus on CHD. Jeong et al. [20] reported a similar finding on the association of triglyceride and CHD but reported a significant association between total cholesterol and CHD which is not the case for total cholesterol in the current study. This could be attributed to the fact that Jeong et al. [20] examined young adults (aged 20-39 years) while JHS participants are relatively older.

Our simulation study suggest that bivariate fitting of such multivariate joint model yields unbiased estimates which are as nearly efficient as the full likelihood model. These results are inline with the performance of the pairwise bivariate fitting in the context of multivariate Gaussian longitudinal data as shown in Fieuws and Verbeke [13] and of semi-continuous longitudinal data given in Kassahun-Yimer et al. [21]. Pairwise bivariate fitting is approximately unbiased in missing completely at random (MCAR) and missing at random (MAR) when outcome-specific parameters are considered, but these results may not necessarily be generalized to all missing data problems [21]. Details on missing data mechanisms can be obtained in Little and Rubin [22]. Alison [23] presented a survey of methods to handle missing data and indicated that maximum likelihood based inference for handling missing data have nearly optimal statistical properties under MAR. Ding and Wang [10], however, showed that in joint modeling of univariate longitudinal response and time-to-event modeling, informative dropout on the longitudinal component could result in serious bias. Multiple imputation is also an attractive approach in handling missing data in some applications despite the several questions posed in relation to the distributional choices, number of imputations and iterations and prior knowledge about the missingness pattern [22, 23]. Fieuws and Verbeke [13] pointed out efficiency loss can be present when some of the parameters are shared by a set of outcomes. An additional simulation conducted to investigate the impact of taking just the baseline values of longitudinal response and including as covariates in survival regression indicated that parameter estimates could be severely biased. This is inline with earlier simulation studies. For example, Henderson et al. [7] and Ibrahim et al. [9] pointed out that severe bias could occur for some parameter estimates when there is ignored latent association between the time-to-event and longitudinal data.

Apart from several advantages, our proposed approach has also limitations. First, right censoring is assumed for the time-to-event outcome, though other mechanisms of censoring could also be operating in practice. Second, we assumed the link between the longitudinal and the survival submodels is constant over time. Third, earlier simulations suggest that pairwise bivariate fitting is approximately unbiased in MCAR and MAR but this may not be the case for all longitudinal data with missingness. Hence, exploring the proposed approach in various missing data mechanisms and patters together with appropriate model diagnostic tools is a useful area of future work.

## Conclusion

In this paper, we have presented and studied a multivariate joint model for multiple longitudinal Gaussian responses and a time-to-event outcome which follows from combining each single joint model of a longitudinal continuous response and the time-to-event outcome via random effects whereby a linear mixed submodel is used for the longitudinal continuous part while a time-to-event submodel is considered for the survival part. The model was illustrated using the Jackson Heart Study. The association between lipid levels and CHD risk obtained from the joint model that takes into account the longitudinal lipid trajectories is substantially higher than that estimated using the baseline lipid values only. The Weibull model (exponential model as its special case) is most common for time-to-event data due to its flexibility, but alternative distributions, such as, the log-normal, log-logistic and gamma could provide better fit to a particular dataset. The choice of one distribution from the other could be based on exploratory graphical tools or formal statistical tests. The pairwise bivariate model fitting approach employed for the Weibull model can be well extended to any other probability density function in the survival submodel.

## Data Availability

Data used in this analysis were produced and used in accordance with the policies of the Jackson Heart Study under contracts from the National, Heart, Lung, and Blood Institute and are not the domain of the authors but that of the Jackson Heart Study. These data are available to other researchers for purposes of reproducing the results or replicating the procedures by submitting a manuscript proposal to the Jackson Heart Study at jhspub *@*umc.edu, as described at https://www.jacksonheartstudy.org/Research/Publications#submitmanuscript. Data updates for the Jackson Heart Study are also deposited regularly in the National Institutes of Health data repositories, dbGaP (https://www.ncbi.nlm.nih.gov/gap/) and BioLincc (https://biolincc.nhlbi.nih.gov/home/).

## Abbreviations

CHD: Coronary heart disease
HDL: High density lipoprotein
JHS: hsCRP: High-sensitivity C-reactive protein
Jackson heart study; SD: Standard deviation
TC: Total cholesterol
TG: Triglyceride
